# Corticospinal Responses Following Gait-Specific Training in Stroke Survivors: A Systematic Review

**DOI:** 10.3390/ijerph192315585

**Published:** 2022-11-24

**Authors:** Yosra Cherni, Alexia Tremblay, Margaux Simon, Floriane Bretheau, Andréanne K. Blanchette, Catherine Mercier

**Affiliations:** 1 Centre Interdisciplinaire de Recherche en Réadaptation et Intégration Sociale, Québec City, QC G1M 2S8, Canada; 2 Département de Réadaptation, Faculté de Médecine, Université Laval, Québec City, QC G1V 0A6, Canada; 3 TOPMED, Centre Collégial de Transfert de Technologie en Orthèses, Prothèses et Équipements Médicaux, Québec City, QC G1S 1C1, Canada

**Keywords:** locomotion, task-oriented training, corticospinal tract, stroke, neuroplasticity

## Abstract

Corticospinal excitability is subject to alterations after stroke. While the reversal of these alterations has been proposed as an underlying mechanism for improved walking capacity after gait-specific training, this has not yet been clearly demonstrated. Therefore, the objective of this review is to evaluate the effect of gait-specific training on corticospinal excitability in stroke survivors. We conducted an electronic database search in four databases (i.e., Medline, Embase, CINAHL and Web of Science) in June 2022. Two authors screened in an independent way all the studies and selected those that investigated the effect of gait-specific training on variables such as motor-evoked potential amplitude, motor threshold, map size, latency, and corticospinal silent period in stroke survivors. Nineteen studies investigating the effect of gait-specific training on corticospinal excitability were included. Some studies showed an increased MEP amplitude (7/16 studies), a decreased latency (5/7studies), a decreased motor threshold (4/8 studies), an increased map size (2/3 studies) and a decreased cortical silent period (1/2 study) after gait-specific training. No change has been reported in terms of short interval intracortical inhibition after training. Five studies did not report any significant effect after gait-specific training on corticospinal excitability. The results of this systematic review suggest that gait-specific training modalities can drive neuroplastic adaptation among stroke survivors. However, given the methodological disparity of the included studies, additional clinical trials of better methodological quality are needed to establish conclusions. The results of this review can therefore be used to develop future studies to better understand the effects of gait-specific training on the central nervous system.

## 1. Introduction

Stroke is a leading cause of physical disability in adults [1]. The prevalence is about 16 million people worldwide [2]. Stroke causes sensorimotor deficits [3,4] that often lead to walking limitations due to the impaired function of neural circuits including the corticospinal tract [5]. It is recognized that the corticospinal tract is the main neural pathway that regulates skilled voluntary movement in humans [6,7]. In this context, studies based on non-invasive brain stimulation techniques, such as transcranial magnetic stimulation (TMS) [8], have reported alterations in corticospinal tract excitability in stroke survivors compared to healthy individuals, such as an increased motor evoked response (MEP) latency [9], an increased resting motor threshold (MT) [10], a reduced MEP size [9] and a prolonged silent period [10]. These alterations in the corticospinal tract contribute to motor performance alterations and are known to be related to gait deficits [11,12,13]. In fact, compared to healthy individuals, post-stroke individuals often exhibit poor motor control ability [14], reduced walking speed [14,15], frequent falls [16], and limited waking endurance [17]. Because gait limitations prevent their independence in daily activities [18,19], a priority for stroke survivors is to optimize gait recovery [20,21].

Gait-specific training interventions, such as overground training [22], treadmill training without or with bodyweight support [23,24] or robotic-assisted gait training [25,26], focus on the automaticity of walking by providing repetitive stepping practice. These modalities have shown several benefits leading to improve walking ability. Systematic reviews [23,27,28] reported that gait-specific training interventions are beneficial to improve functional/clinical parameters of gait (e.g., walking speed, walking endurance, and gross motor function) in individuals with neurological disorders. The functional gains resulting from gait-specific training in stroke survivors, like those produced by gait-specific training, may be due to several mechanisms, such as re-establishing control performed by ipsilesional sensorimotor cortex [29,30] and behavioral compensation strategies [31]. In animals and humans, some studies provided evidence of a change in activation patterns in many regions of the damaged brain [32,33]. Changes in corticospinal excitability might reflect a contribution of primary motor cortex reorganization in functional gains [34,35]. However, although several reviews investigated the effect of interventions on walking capacity in stroke survivors, their impact on corticospinal excitability remains to be clearly established. Therefore, the objective of the present literature review was to summarize and evaluate the effect of gait-specific training on corticospinal excitability in post-stroke individuals.

## 2. Materials and Methods

### 2.1. Data Source and Literature Source

The search protocol was developed by the authors and validated by a science librarian. Studies were identified by searching in 4 databases (i.e., Medline, Embase, CINHAL and Web of Science) from inception to June 2022. The search strategy was based on three main concepts: gait-specific training, corticospinal excitability, and stroke population. More details concerning the search strategy and the keywords used are reported in Table S1 as Supplementary Materials. The current study respects the guidelines for the preferred reporting items for systematic reviews (PRISMA) [36] and was registered in the PROSPERO register on 21 June 2022 (ID: CRD42022338555).

### 2.2. Eligibility Criteria

The included studies were included if they met the following inclusion criteria: (1) intervention studies, such as randomized controlled trials (RCT), pre/post studies, case studies, etc.; (2) studies targeting people with poststroke; (3) studies based on gait-specific training modality that focus on practicing tasks related to gait (e.g., overground gait training, treadmill training, robotic assisted gait training, etc.); (4) studies reporting at least one variable related to corticospinal excitability measured with TMS (i.e., MEP, MT, map size, latency, and cortical silent period); (5) studies evaluating pre/post effect of the intervention on corticospinal excitability and; (6) studies published in French or English. 

We excluded studies if they: (1) were performed in a mixed population without a possibility to isolate results from individuals who have suffered a stroke; (2) were based on multiple training modalities (e.g., including repetitive TMS, transcranial direct-current stimulation, etc.) among which we cannot distinguish the effects of gait-specific training on corticospinal excitability and (3) were not original research (e.g., commentaries, letters to the editor, etc.).

### 2.3. Screening of Studies

Titles and abstracts were screened independently by two of the authors (Y.C. and A.T.) to identify those that potentially met the inclusion criteria. A full review of those papers was then performed independently by the same authors. Article selection was discussed until consensus was reached. In the case of any unresolvable disagreement related to the studies eligibility, a third author (C.M.) intervened to make a decision.

### 2.4. Methodological Quality and Risk of Bias

To assess the methodological quality of included studies, two checklists were used in this study. First, YC and AT independently rated the overall quality of each included article, using the PEDro scale [37]. The PEDro scale consists of 11 items [37]: **Item 1.** eligibility criteria were specified. **Item 2.** subjects were randomly allocated to groups. **Item 3.** allocation was concealed. **Item 4.** The groups were similar at baseline regarding the most important prognostic indicators. **Items 5–7.** There was blinding of all subjects, therapists, and assessors. **Item 8.** Measures of at least one key outcome were obtained from more than 85% of the subjects initially assigned to groups. **Item 9.** All subjects for whom outcome measures were available received the treatment or control condition assigned or, where this was not the case, data for at least one key outcome were analyzed by “intention-to-treat”. **Item 10.** The results of between-group statistical comparisons are reported for at least one key outcome. **Item 11.** The study provides both point measures and measures of variability for at least one key outcome. Each item is scored as a “yes” or “no”, worth 1 or 0 points, respectively. The total score expressed on a 10-point scale. The first item is not included in the sum on the total score of the PEDro scale. A PEDro score is considered poor between 0 and 3, fair between 4 and 5, good between 6 and 8, and excellent between 9 and 10 [38].

Second, the Chipchase checklist was used to evaluate the methodology and reporting of studies in relation to the use of TMS [39]. In this checklist, 8 items are related to subjects (e.g., age, gender) and 18 to methodology (e.g., coil type, stimulus intensity, etc.). the items consist of [39]: **Item 1:** Age of subjects; **Item 2:** Gender of subjects; **Item 3:** Handedness of subjects; **Item 4:** Subjects prescribed medication; **Item 5:** Use of central nervous system active drugs (e.g., anti-convulsant); **Item 6:** Presence of neurological/psychiatric disorders; **Item 7:** Any medical conditions; **Item 8:** History of specific repetitive motor activity. **Item 9:** Position and contact of EMG electrodes; **Item 10:** Amount of contraction of target muscles; **Item 11:** Prior motor activity of the muscle to be tested; **Item 12:** Relaxation of muscles other than those tested; **Item 13:** Coil type (size and geometry); **Item 14:** Coil orientation; **Item 15:** Direction of induced current in the brain; **Item 16:** Coil location and stability; **Item 17:** Type of stimulator used (e.g., brand); **Item 18:** Stimulation intensity; **Item 19:** Pulse shape (monophasic or biphasic); **Item 20:** Determination of optimal hotspot, as defined by the area on the scalp where the TMS produces the largest MEP size [8]; **Item 21:** The time between MEP trials; **Item 22:** Time between days of testing; **Item 23:** Subject attention (level of arousal) during testing; **Item 24:** Method for determining threshold (active/resting); **Item 25:** Number of MEP measures made; **Item 26:** Method for determining MEP size during analysis.

For both assessment procedures, a first meeting was initially held with five articles, to ensure a clear understanding of each criterion and thus standardization and reliability of assessments. A second meeting was held to discuss the criteria for each included article, until a consensus was reached about the score. In the case of any disagreement, a third author (C.M.) performed the assessment to make a decision. 

### 2.5. Data Extraction

Data including study design, quality assessment, participants characteristics, intervention (and comparison with a control group), outcomes, and results, were extracted by one author (M.S.) and validated by a second author (Y.C.). Outcomes of interest were measurements of corticospinal excitability such as MEP size, MEP latency, TMS map area, MT, cortical silent period and short interval intracortical inhibition Readers who are not familiar with these measurements can refer to [8] for a description of these variables and a discussion of underlying neurophysiological mechanisms. In studies in which the gait-specific intervention was a control condition (the experimental condition being for example gait training combined with brain stimulation), the data were extracted only for the pre/post effect of this condition. The quality rating was performed based on a pre/post in such case, therefore reflecting the quality of study based on the data extracted in response to the review objective, and not the quality of the original study.

## 3. Results

### 3.1. Search Results

**Figure 1:**
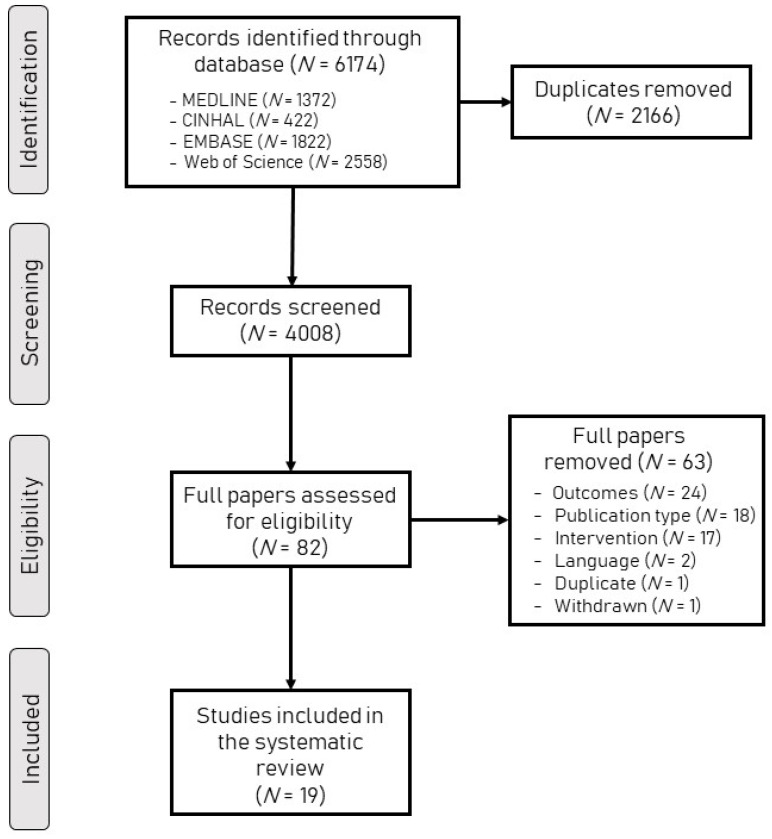
The flowchart presenting the protocol of this systematic review.

The search and the screening processes are summarized in Figure 1. The initial search identified 6174 articles. After removing duplicates, the eligibility of 4008 articles was independently evaluated by two reviewers based on their titles and abstracts. In this process, 82 articles were determined by consensus to qualify for the full-text reading stage. This last stage resulted in the identification of 19 articles as eligible in this review. 

### 3.2. Risk of Bias

Study design and quality assessment: Table 1 summarizes the PEDro rating score for each of the 19 studies, which included 6 RCTs [35,40,41,42,43,44], 2 crossover studies [45,46], 1 cross-sectional study [34], 8 pre/post studies [47,48,49,50,51,52,53] and 2 cases studies [54,55]. The methodological quality of the included studies ranged from 1 to 8 out of 10, with a median score of 4. Nine studies were of high quality (PEDro score ≥ 6), six studies were of moderate quality (PEDro score = 4–5) and four were of poor quality (PEDro score ≤ 3). In six studies [47,48,49,50,52,53], the gait-specific training was considered a control condition to another modality such as Transcranial direct current stimulation or repetitive TMS. To meet the objective of this review, data extraction in these studies only concerned the pre/post effect of gait-specific training interventions (see Table 1).

TMS methodological quality: The specific details of the included studies, which incorporate the evaluation of the Chipchase checklist, are summarized in Table 2. The included studies had scores ranging from 5 to 20 out of 26, with a median score of 14. Regarding participant factors, one study reported prescribed subject medication (**Item 4**), 17 studies reported the use of central nervous system active drugs (**Item 5**); and five studies described participants medical condition (**Item 7**). The use of medications acting on the central nervous system or impacting TMS measures was considered an exclusion criterion in 17/19 studies and two studies did not report any information on medication. Concerning methodological factors, the majority of studies reported coil location and stability (*N* = 14), current direction (*N* = 7) or method for determining MEP size during analysis (*N* = 12). Only three studies described stimulation pulse shape (*N* = 3) and no studies controlled the level of relaxation present in the muscles other than those being tested.

### 3.3. Characteristics of the Participants

The sample size in the included studies ranged from 1 to 50 participants (total of 362 across all studies), and participant demographics varied considerably (see Table 3). Sixteen studies focused on participants in the chronic phase of recovery (>3 months post-lesion) after stroke, three studies [41,43,49] included participants in the subacute phase and one study [55] included one participant in the acute phase. 

### 3.4. Gait Training Protocols

Training parameters (modalities, frequency, session duration and total number of sessions) are displayed in Table 3. The protocols of the included studies were heterogeneous (e.g., duration: 1—24 weeks; frequency: 1—5 sessions/week). Of the five studies proposing 1-training session, only two studies showed positive effects following gait-specific training (High intensity interval treadmill training [46] or walking with functional electrical stimulation [45]). However, the majority of studies reporting positive effects were based on protocols with higher training volume (≥12 sessions). 

### 3.5. Effect of Gait-Specific Training on Corticospinal Excitability

**Figure 2:**
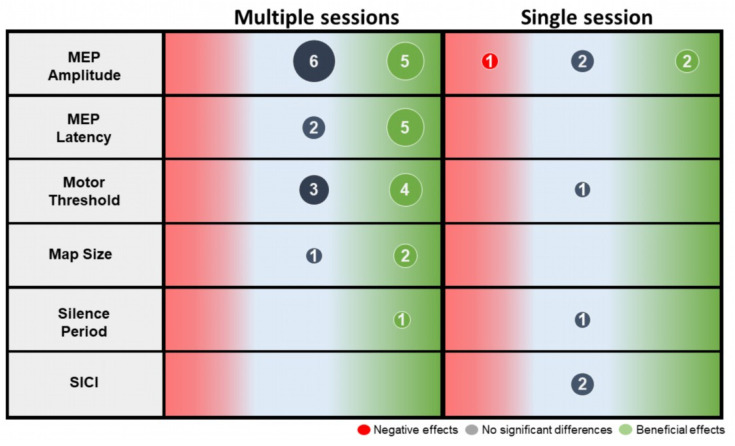
Synthesis of the pre-post effect of single and multiple gait training sessions on corticospinal excitability by reporting the number of articles showing a positive effect (green), no effect (grey) or a negative effect (red). Numbers represent the number of articles. Abbreviations: MEP: motor evoked response, SICI: Short interval intracortical inhibition.

The results extracted from the included studies are summarized in Figure 2 and Table 3. The following sections outline the effect of gait-specific training on corticospinal excitability in terms of MEP amplitude and latency, motor threshold, map size, cortical silent period, and short interval intracortical inhibition.

MEP amplitude: Sixteen studies have investigated the effect of gait-specific training on MEP amplitudes. Seven studies showed a significant increase in MEP amplitudes after gait training (i.e., robotic training [40,42,43,44], treadmill training [46], overground training [49] and functional electrical stimulation combined to overground training [12]). Five of these studies [40,42,43,44,45] presented high methodological quality and two [46,49] were of moderate quality. However, eight studies did not report significant change [34,47,48,50,51,53,55,56] and one study showed a decrease in MEP amplitudes after gait-specific training [54]. Six of these seven studies were of moderate to low methodological quality. Only one study (case study, PEDro = 2) reported a negative effect of gait training on MEP amplitude [54]. In general, an increase in the amplitude of MEP may result from gait-specific training; however, in order to better assess corticospinal excitability other TMS parameters should also be considered to compensate for the variability that may be present in the MEP results.

MEP latency: Seven studies investigated the effect of gait-specific training on MEP latency. Data from the five studies indicated a decrease in response to conventional gait training [49], robotic training [43,44,49,56], treadmill training [34] and body weight support treadmill training [44]. Three of these studies were RCTs (PEDro = 6–7) and two were pre/post studies (PEDro = 2–4). On the other hand, two pre/post studies (PEDro = 3–4) did not observe a significant change in MEP latency after training [47,50].

Motor threshold: MT was reported in eight studies. Four studies reported a decrease in MT after gait-specific training (i.e., overground gait training [55], body weight support treadmill training [35,41] and robotic gait training [44]), while the other four studies [34,50,51,52] did not report any significant changes after training. Three of the four studies showing a positive effect of gait-specific training on MT are RCT (PEDro < 7), while those indicating a lack of change are pre/post studies (PEDro = 3–4). In general, a significant decrease in MT was observed after robotic training [44,50] or body weight support treadmill training [35,41,44]. Moreover, Yang et al. [41] observed a decrease in the MT in subacute group but not in the chronic group after body weight support treadmill training.

Map size: Three studies used the TMS mapping technique to estimate the effect of gait-specific training on the size of the corticomotor representation [35,41,56]. Two RCT studies (PEDro = 7–8) reported an increase in map size after body weight support treadmill training [35,41]. Furthermore, in their pre/post study (PEDro = 2), Poydasheva et al. [56] did not observe a significant change in this same parameter. Yang et al. [41] found that this increase in map size after gait training was greater in subacute patients compared to chronic patients. Although the results of the two RCTs appear promising in terms of increase in the size of the map, the sample size of these studies remains limited (≤7 participant per group) which limits the conclusions on this variable.

Cortical silent period duration: Two studies examined the effect of gait-specific training on the cortical silent period. One study (PEDro = 1) showed a decrease in the cortical silent period after overground training in one participant [55] while the other (PEDro = 3) did not observe any change in a group of 12 participants [51]. Although the study reporting a lack of effect had a larger sample size, it is important to bear in mind that it looked at the effect of a single training session. Therefore, the available data are too limited to reach a conclusion on this variable. 

Short interval intracortical inhibition: Two studies [46,51] explored the effect of 1-session gait training on short interval intracortical inhibition in 12–13 stroke survivors. No significant changes were observed after training.

## 4. Discussion

This review summarized how responses to a single or multiple sessions of gait-specific training can modulate corticospinal excitability in stroke survivors. In general, gait-specific training may enhance corticospinal excitability in stroke survivors. However, given the moderate number of RCT and crossover studies and the overall methodological disparity of included studies (PEDro = 1–8), further clinical trials with higher quality designs are needed to better understand the corticospinal responses to gait-specific training.

### 4.1. Effect of Gait-Specific Training on Corticospinal Excitability

An effective rehabilitation intervention can modulate the way the brain controls movement [57]. Previous studies showed a remapping of movement representations in M1 in animals after effective rehabilitative training of hand movement after a brain injury [58,59]. Reorganization of corticospinal actions by gait-specific training in individuals with neurologic diseases has been shown in previous studies [58,60]. In this systematic review, we focused on the effect of gait-specific training on corticospinal excitability in stroke survivors on TMS-related outcomes. Most of the included studies (16/19) targeted MEP amplitude, which is an indicator of corticospinal excitation [6]. Among these studies, seven studies showed a significant increase in MEP amplitudes after different modalities of gait-specific training (i.e., robotic training [40,42,43,44], treadmill training [46], overground training [49] and functional electrical stimulation combined to overground training [45]). Furthermore, the MEP increases after training have been observed in rectus femoris [42,44], tibialis anterior [43,44,46], soleus [45], medial gastrocnemius [44], hallux abductor [40] and extensor carpi radialis [46] muscles. However, it is important to mention that eight studies did not report significant change [34,47,48,50,51,53,55,56] and one study [54] showed a decrease in MEP amplitudes after gait-specific training. These studies were mostly of low and moderate methodological quality and performed in participants at the chronic stage. Finally, changes in MEP amplitude have been often investigated as it is relatively easy to quantify. However, other TMS variables might offer a better reliability, such as MT and latency [61].

Lower MTs are associated with increased M1 excitability [62]. Interventions such as motor skill training have been shown to reduce MT in humans [63]. In the present review, several clinical trials reported a reduced MT after gait-specific training (e.g., robotic training [44] or body weight support treadmill training [35,41,44]) in stroke survivors. An important finding was also the reduction in MEP latency after task-specific gait training [34,43,44,49,56]. This variable appears to be an indicator of lower limb impairment and walking limitations [64]. In two RCTs, the authors reported an increase in map size after body weight support treadmill training [35,41]. This allows us to conclude that body weight support treadmill training may enlarge the cortical motor representation of tibialis anterior and hallux abductor muscles. Furthermore, a case study [55] showed a decrease in the cortical silent period after 12 sessions of overground training, while the other [51] did not observe any change after one training session in a group of 12 participants. Given the sample size and the methodological quality of these studies, it is difficult to draw conclusions on this variable. In conclusion, results derived from several studies on the effect of gait-specific training suggest a positive effect on corticospinal excitability in stroke survivors. However, the lack of consistency in the results, the methodological disparity of included studies (e.g., differences assessed muscles, intervention durations, etc.) and the methodological shortcomings in the TMS use should be considered. 

It is important to emphasize that some studies included in this review [47,48,50,51,52,54] did not show a significant increase in corticospinal excitability after gait-specific training in stroke survivors. The methodological shortcomings in the use of TMS could explain the disparity in the results of corticospinal excitability [65]. For example, the hot spot is not always well defined and is sometimes optimized for one muscle while the study evaluates several muscles. The lack of significant post-training change may also be due to the protocols of these studies which are based on a low training volume (e.g., 1–12 sessions) [66]. It is possible that an initial increase in corticospinal excitability may still increase with several days and weeks of training but will eventually stagnate and decrease as training progresses without additional challenge [67]. Evidence suggested that the efficacy of post-stroke motor rehabilitation is related to the degree to which the neuromuscular system is challenged by repetitive voluntary movement [68,69]. A single gait-specific training session performed was not sufficient to induce short-term effects on corticospinal excitability parameters [51,53,54] in stroke survivors, except for MEP amplitudes [45,46]. One important methodological factor to consider is that in these studies, measurements were taken right after the training session, while they were typically taken on a different day in studies with multiple sessions. Results of studies using a single session might therefore be impacted by factors such as muscle pre-activation or muscle fatigue. Overall, training parameters (e.g., intensity, session duration, frequency) need to be decided in an objective manner [70]. 

### 4.2. Clinical Recommendations

Despite the methodological disparity of the included studies, some clinical recommendations can be derived from this review. Mostly, studies showed changes in corticospinal excitability after high training volume (≥12 sessions) of gait-specific training in stroke survivors. This observation is consistent with the previous recommendations [66,71] that higher training intensities and durations may promote brain plasticity. However, the heterogeneity of samples and the variability of training modalities, frequencies, durations, and intensity complicate the generation of clear recommendation for optimal gait training parameters that enhance corticospinal excitability after stroke. Furthermore, only one study [41] with a small sample size investigated the effect of gait-specific training on corticospinal excitability in relation to stroke duration; the authors observed a greater increase in corticospinal excitability in the subacute group than the chronic group after body weight support treadmill training. On the other hand, the studies [47,48,50,51,52,53] that did not report significant changes were those targeting individuals in chronic phase. This observation supports the recommendations to start rehabilitation as soon as possible after a stroke [72].

Finally, TMS is a valid tool to evaluate the corticospinal excitability, but it is a technique that presents intra-subject variability and there are few studies on the lower limbs and even less during walking; it is therefore important to be more rigorous in its use, in particular by using TMS-specific components checklist [39]. In our review, we found that the results of the PEDro evaluation did not match the Chipchase checklist. Thus, studies with a high PEDro rating score are not necessarily of good quality from TMS methodology perspective. In conclusion, clinical trials with better methodological quality are needed to better understand the corticospinal responses to gait-specific training.

### 4.3. Limitations

Some limitations in this review must be acknowledged. First, we reported only TMS outcomes to understand the effect of gait-specific training on corticospinal excitability, while other variables such as EMG-EMG or EEG-EMG coherence might also offer some relevant insight [73]. This choice was made to limit the heterogeneity of the results and allow methodological comparisons across studies. Second, the included studies were diverse regarding the population of patients with stroke, especially regarding the wide variation in time since stroke. Third, another limitation of this review concerns restrictions of publication language and type of publication; therefore, a publication bias might be present.

## 5. Conclusions

This review is a first step towards understanding how the corticospinal pathway responds to a single or multiple sessions of gait-specific training. Overall, the results suggest that multiple gait-specific training modalities can drive neuroplastic adaptation among post-stroke survivors even in a chronic phase of recovery. Future studies should aim for higher-quality designs and better TMS methodology so that clear recommendations can emerge and be applied in stroke rehabilitation.

## Figures and Tables

**Table 1:** Study design and quality assessment (PEDro score) of the included studies.

Authors	Design	Item 1	Item 2	Item 3	Item 4	Item 5	Item 6	Item 7	Item 8	Item 9	Item 10	Item 11	Total/10
**Calabrò** [40]	RCT	1	1	1	1	0	0	1	1	1	1	1	**8**
**Yang** [41]	RCT	1	1	1	1	0	0	1	1	1	1	1	**8**
**Jayaraman** [42]	RCT	1	1	0	1	0	0	1	1	1	1	1	**7**
**Li** [43]	RCT	1	1	0	1	0	0	1	1	1	1	1	**7**
**Shahine** [44]	RCT	1	1	0	1	0	0	1	1	1	1	1	**7**
**Yen** [35]	RCT	1	1	1	1	0	0	0	1	1	1	1	**7**
**Forrester** [34]	Cross sectional	1	1	0	1	0	0	0	1	1	1	1	**6**
**Palmer** [45]	Crossover study	1	1	0	1	0	0	0	1	1	1	1	**6**
**Li** [46]	Crossover study	1	1	0	1	0	0	0	1	1	0	1	**5**
**Wang** [47] *	Pre/Post study *	1	0	1	0	0	0	0	1	1	0	1	**4**
**Chang** [49] *	Pre/Post study *	1	0	0	0	0	0	0	1	1	0	1	**3**
**Seo** [50] *	Pre/Post study *	1	0	0	0	0	0	0	1	1	0	1	**3**
**Wang** [48] *	Pre/Post study *	1	0	0	0	0	0	0	1	1	0	1	**3**
**Wong** [51]	Pre/Post study *	1	0	0	0	0	0	0	1	1	0	1	**3**
**Koganemaru** [52] *	Pre/Post study *	1	0	0	0	0	0	0	1	1	0	1	**3**
**Madhavan** [53] *	Pre/Post study *	1	0	0	0	0	0	0	1	1	0	1	**3**
**Poydasheva** [56]	Pre/Post study	1	0	0	0	0	0	0	1	1	0	0	**2**
**Krishnan** [54]	Case study	0	0	0	0	0	0	0	0	1	0	1	**2**
**Peurala** [55]	Case study	0	0	0	0	0	0	0	0	1	0	0	**1**

NOTE. RCT: Randomized Controlled Trial. A PEDro score is considered poor between 0 and 3, fair between 4 and 5, good between 6 and 8, and excellent between 9 and 10 [38]. * These studies were RCT [48,49,50,51] *or crossover studies* [52,53]. However, in the present review, they were considered as Pre/Post studies as only data from the control group were extracted in relation to the objective of the review. Items were rated accordingly, and therefore the score do not reflect the overall quality of the original study.

**Table 2:** Please TMS-specific components of methodological quality using the Chipchase checklist.

Studies	Participant Factors								Methodological Factors																		Total
	**1**	**2**	**3**	**4**	**5**	**6**	**7**	**8**	**9**	**10**	**11**	**12**	**13**	**14**	**15**	**16**	**17**	**18**	**19**	**20**	**21**	**22**	**23**	**24**	**25**	**26**	**/26**
**Calabrò** [40]	1	1	1	0	1	1	1	0	0	1	0	0	1	0	0	0	1	0	1	0	0	1	0	0	1	1	**13**
**Yang** [41]	1	1	1	0	1	0	0	0	0	1	0	0	1	1	0	1	1	1	0	1	0	1	0	1	0	1	**14**
**Jayaraman** [42]	1	1	1	0	1	1	1	0	1	1	1	0	1	1	1	0	1	1	0	1	1	1	0	1	1	1	**20**
**Li** [43]	1	1	1	1	1	1	0	0	1	1	0	0	1	1	1	1	1	1	0	1	0	1	0	1	1	1	**19**
**Shahine** [44]	1	1	1	0	1	1	1	0	0	1	0	0	1	0	0	1	1	0	0	0	0	1	0	1	0	1	**13**
**Yen** [35]	1	1	1	0	1	1	0	0	0	1	0	0	1	1	0	1	1	1	0	0	0	1	0	1	0	0	**13**
**Forrester** [34]	1	1	1	0	1	1	1	0	1	1	1	0	1	1	0	1	1	1	0	0	0	1	0	1	1	1	**17**
**Palmer** [45]	1	1	0	0	1	1	0	0	1	1	1	0	1	1	1	0	0	0	1	0	1	1	1	1	1	1	**17**
**Li** [46]	1	1	1	0	1	1	0	0	1	0	0	0	1	0	0	1	1	1	0	1	0	1	0	1	0	1	**14**
**Chang** [49]	1	1	1	0	1	1	0	0	1	0	0	0	1	0	0	1	1	1	0	1	1	1	0	1	1	1	**16**
**Seo** [50]	1	1	1	0	1	1	1	0	0	1	0	0	1	0	0	0	1	0	0	1	0	1	0	0	0	1	**12**
**Wang** [47]	1	1	1	0	1	1	0	0	0	1	0	0	1	1	1	1	1	1	0	0	1	1	0	1	1	1	**17**
**Wang** [48]	1	1	1	0	1	1	0	0	1	1	0	0	1	0	0	0	1	1	0	1	0	1	0	1	0	1	**14**
**Wong** [51]	1	1	1	0	1	1	0	0	0	1	1	0	1	1	1	1	1	1	0	0	0	1	0	0	0	1	**15**
**Koganemaru** [52]	1	1	0	0	1	1	0	0	1	1	1	0	1	1	1	1	1	0	0	0	0	1	0	1	0	0	**14**
**Madhavan** [53]	1	1	1	0	1	1	0	0	1	1	1	0	1	1	1	1	1	0	0	0	0	1	1	1	0	1	**17**
**Poydasheva** [56]	1	0	1	0	0	0	0	0	0	0	0	0	0	0	0	1	1	0	0	0	0	1	0	0	0	0	**5**
**Krishnan** [54]	1	1	1	0	0	1	0	0	1	0	0	0	1	1	0	1	1	0	0	0	0	1	0	1	1	0	**12**
**Peurala** [55]	1	1	1	0	1	1	0	0	1	1	1	0	1	0	0	1	1	0	1	1	0	1	0	1	0	0	**15**

**Table 3:** Summary of studies: populations, interventions, outcomes and results.

Authors	*N*	Age (Years) (Mean ± SD or Median [Quartiles])	Time Poststroke (Mean ± SD or Median [Quartiles])	Interventions		Key Outcomes	Results
				Experimental	Control		
**Calabrò** [40]	EG = 20 CG = 20	EG = 69 ± 4 CG = 67 ± 6	EG = 10 ± 3 months CG = 11 ± 3 months	Exoskeleton (Ekso) training	Conventional overground gait training	MEP amplitudes (peak to peak) in HA in both sides. Stim intensity: N/S	**In both groups**: ↑ MEP amplitude on the paretic side. Greater change **in EG**. ↓ MEP amplitude in non-paretic side in **EG**.
				40 sessions: 8weeks—5x/week			
**Chang** [49]	EG = 12	EG = 59.9 ± 10.2	EG = 16.0 ± 6.2 days	Overground gait training including postural control, motor function, and movement patterns.	None	MEP amplitudes (peak to peak) and latency in TA in affected side at rest. Stim intensity: 100% MSO	↑ MEP amplitude (+127%) and ↓ MEP latency (−3%) after training.
				10 sessions: 2 weeks—5x/week			
**Forrester** [34]	EG = 3 CG = 8	EG = 65.3 ± 6.3 CG = 62.2 ± 1.7	EG = 31.2 ± 20.4 months CG = 31.2 ± 20.4 months	A group previously trained with treadmill received a submaximal effort (60% heart rate reserve) treadmill training.	A non-trained treadmill group received a submaximal effort (60% heart rate reserve) treadmill training.	RMT, MEP amplitudes (peak to peak) and latency in VM on both sides at rest. Stim intensity: 110% RMT	**In CG**: ↓ MEP latencies in the paretic and non-paretic side (−7%). No significant change in the **EG**. No significant difference between groups.
				72 sessions: 24 weeks—3x/week			
**Jayaraman** [42]	EG = 25 CG = 25	EG = 59.5 ± 9.7 CG = 61.6 ± 12.6	EG = 85.2 ± 74.4 months CG = 64.8 ± 36.0 months	Exoskeleton (Honda Stride Management Assist) training	Treadmill gait training + patients’ goals-oriented tasks	MEP amplitudes (Slope) in RF, MH and TA in both sides. Stim intensity: Recruitment curves for each muscle were obtained by collecting MEPs for a range of stimulus intensities from 80% to 140% of AMT, in increments of 10%, resulting in 7 total intensities.	**In both groups**: ↑ MEP amplitudes of paretic RF. Greater change in **EG** (+178% ± 75%) vs. in **CG** (33% ± 32%). **In CG**: ↑ MEP amplitude in MH (110%) and TA (214%).
				18 sessions: 6–8 weeks s; 3x/week			
**Koganemaru** [52]	N = 11	65.7 ± 3.6	74.4 ± 32.3 months	Gait training on a treadmill and FES to assist paretic ankle.	None	AMT in TA and gastrocnemius muscles in both sides. Stim intensity: N/S	No significant differences.
				12 sessions: 4 weeks—3x/week			
**Krishnan** [54]	N = 1	52	7.0 months	RAGT (Lokomat)	None	MEP amplitudes during gait in GM, RF, VM, MG, SOL (during stance) MH, LH, TA (during swing). Stim intensity: N/S	↓ MEP amplitude of the VM (−55%), MH (−72%) and GM (−66%) muscles after training.
				1 session			
**Li** [46]	N = 13	65.8 ± 7.2	39.5 ± 33.7 months	**Phase 1:** high-intensity exercise priming (i.e., fast treadmill walking); **Phase 2:** rest	None	MEP amplitudes (peak to peak) in extensor carpi radialis and SICI in both sides. Stim intensity: 120% RMT	↑ MEP post-exercise in paretic side compared to rest in paretic side (+0.35%).
				1 session			
**Li** [43]	EG = 12 CG = 13	EG = 51.2 ± 7.8 CG = 49.6 ± 8.4	N/A	Exoskeleton (BCI-LLRR) + routine rehabilitation	Routine rehabilitation interventions (pulsed electrical therapy, partial hemiplegia comprehensive training)	MEP amplitudes (peak to peak) and latency in TA in both sides. Stim intensity: 90% TA muscle AMT.	**In both groups**: ↓ MEP latencies and ↑ MEP amplitude. Greater change in **EG**.
				30 sessions: around 4 weeks			
**Madhavan** [53]	N = 11	58 ± 2.7	108.0 ± 21.6 months	High intensity interval treadmill training.	None	MEP amplitudes (peak to peak) in TA in both sides. Stim intensity: N/S	No significant differences.
				1 session			
**Palmer** [45]	N = 20	59.5 ± 12.0	42.0 ± 2.05 months	**Phase 1:** Walking with FES; **Phase 2:** Walking without FES.	None	MEPs amplitudes in TA and SOL in both sides. Stim intensity: N/S	↑ MEP amplitudes in the paretic SOL (+30%) following gait training with FES.
				1 session/intervention—1week apart			
**Peurala** [55]	N = 1	76.0	Acute phase	Conventional gait training (standing and overground exercises)	None	RMT, MEP amplitudes and silent period in TA in both sides. Stim intensity: N/S	↓ RMT and silent period in the non-paretic side.
				15 sessions: 3 weeks—5x/week			
**Poydasheva** [56]	N = 14	53.0 yrs [49.0; 62.0];	14.2 [7.0; 2.0] months	Standard rehabilitation + rehabilitation exercises with Exoskeleton	None	MEP amplitudes and latency in TA and map size in both sides. Stim intensity: N/S	↓ MEP latency (−8.5%) in the paretic side.
				10 sessions: 2 weeks s—5x/week			
**Seo** [50]	N = 10	62.9 ± 8.9	152.5 ± 122.8 months	RAGT on a treadmill (Walkbot_S) + sham tDCS	None	RMT, MEP amplitudes (peak to peak) and latency in HA in both sides. Stim intensity: N/S	No significant differences.
				10 sessions: 2 weeks—5x/week			
**Shahine** [44]	EG = 25 CG = 25	EG = 58.3 ± 8.6 CG = 59.7 ± 7.4	EG = 30.3 ± 21.8 months CG = 28.4 ± 19.8 months	Electromechanical gait training (GT): movements of lower limb are assisted.	BWSTT: weight support with free movements of lower limb.	RMT, MEP amplitudes (peak to peak) and latency in RF, TA, MG. Stim intensity: N/S	**In both groups**: ↓ RMT in RF, TA, MG; ↑ MEP amplitude in RF, TA, MG; ↓ MEP latency in RF, TA, MG. No significant differences between groups.
				48 sessions: 8 weeks—6x/week			
**Wang** [47]	N = 12	62.9 ± 10.9	24.0 ± 14.0 months	Sham rTMS, followed by functional task-oriented training (standing and walking)	None	MEP amplitudes (peak to peak) and latency in RF sides at rest. Stim intensity: 110% of RMT	No significant differences.
				10 sessions: 2 weeks—5x/week			
**Wang** [48]	N = 6	54.7 ± 12.2	31.8 ± 24 months	Regular physical therapy + Sham rTMS, followed by treadmill training	None	MEP amplitudes (peak to peak) in TA in both sides at rest. Stim intensity: 120% of RMT	No significant differences.
				9 sessions: 3 weeks—3x/week			
**Wong** [51]	N = 12	57.3 [46.1; 62.8]	54.0 [24.0; 93.4] months	Walking under 3 conditions: cognitive dual task walking, motor dual task walking, and single walking.	None	RMT, MEP amplitudes (peak to peak), cortical silent period duration, and SICI in the paretic TA during contraction. Stim intensity: 120% RMT	No significant differences.
				1 session of 20 min each exercise			
**Yang** [41]	EG _Chro_ = 5 EG _Sub_ = 5 CG _Chro_ = 4 CG _Sub_ = 4	EG _Chro_ = 57.5 ± 6.1 EG _Sub_ = 56.8 ± 1.3 CG _Chro_ = 48.1 ± 3.7 CG _Sub_ = 61.8 ± 3.8	EG _Chro_ = 25.2 ± 3.6 months EG _Sub_ = 3.0 ± 1.0 months CG _Chro_ = 34.8 ± 6.0 months CG _Sub_ = 3.0 ± 1.0 months	BWSTT + General exercise program (stretching, strengthening, endurance, overground walking training)	General exercise program (stretching, strengthening, endurance, overground walking training)	RMT and map size of HA at rest. Stim intensity: 110% of RMT	**In EG**: ↓ RMT in subacute patients (−23%). ↑ map size in subacute (+134%) and chronic patients (+38%).
				12 sessions: 4 week—3x/week			
**Yen** [35]	EG = 7 CG = 7	EG = 57.3 ± 16.4 CG = 56.0 ± 12.7	EG = 22.8 ± 7.32 months CG = 22.8 ± 7.32 months	BWSTT + General exercise program (stretching, strengthening, endurance, overground walking training)	General exercise program (stretching, strengthening, endurance, overground walking training)	RMT, map size of TA and HA in both sides at rest. Stim intensity: 110% of RMT	**In EG**: ↓ RMT for TA in the non-paretic side (−9%). ↑ map size for TA in the paretic (+24%) and non-paretic (+35%) sides. ↑ map size of AH in the paretic side (500%).
				12 sessions: 3x/week of BWSTT; 2 to 5x/week of general exercise.			

Abbreviations: EG: Experimental group; CG: Control group; N/S: Non-specified; PS: paretic side; NPS: non-paretic side; MEP: Motor evoked potential; HA: hallux abductor; TA: Tibialis anterior; VM: vastus medialis, RF: rectus femoris, MH: medial hamstrings, LH: lateral hamstrings, MG: medial gastrocnemius, SOL: soleus, GM: gluteus medius; MSO: Maximum stimulator intensity; RMT: resting motor threshold; AMT: active motor threshold; RAGT: Robot-assisted gait training; BCI-LLRR: brain-computer interface-operated lower limb rehabilitation robot; Sham rTMS: sham repetitive transcranial magnetic stimulation; tDCS: transcranial direct-current stimulation; BWSTT: Body weight-supported treadmill training; FES: Functional Electrical Stimulation; Chro: chronic; Sub: Subacute.

## Data Availability

Not applicable.

## Supplementary Materials

The following supporting information can be downloaded at: https://www.mdpi.com/article/10.3390/ijerph192315585/s1, Table S1: Research strategy.
